# Parallel Analysis of Acidic and Basic Proteoforms in Cell Lysates via Native Cation and Anion Exchange Chromatography—Native Mass Spectrometry

**DOI:** 10.1002/pmic.70134

**Published:** 2026-04-21

**Authors:** Ziran Zhai, Hafsa Zakri, Matteo Damian, Francesco G. Mutti, Garry Corthals, Andrea F. G. Gargano

**Affiliations:** ^1^ Analytical Chemistry Group and Biocatalysis Group, Van't Hoff Institute For Molecular Sciences (HIMS) University of Amsterdam Amsterdam The Netherlands; ^2^ Centre For Analytical Sciences Amsterdam, Van't Hoff Institute For Molecular Sciences (HIMS) University of Amsterdam Amsterdam The Netherlands

**Keywords:** anion exchange chromatography, cation exchange chromatography, intact proteins, nanoflow rate, native mass spectrometry

## Abstract

Comprehensive characterization of proteoforms in complex biological systems remains a significant challenge. While native ion‐exchange chromatography (IEC) hyphenated with native mass spectrometry (nMS) is a powerful tool for resolving proteoforms, three critical limitations persist: restricted volatile buffers causing nonlinear pH gradients; insufficient coverage of the proteome's broad isoelectric point (pI) range; and inadequate sensitivity for low‐abundance proteins. To bridge this gap, we developed an online nanoflow dual IEC‐nMS platform. Volatile salt systems for strong anion (SAX) and cation exchange (SCX) were optimized to achieve exceptionally wide and linear pH ranges (2.6–5.0 for SAX and 5.0–8.5 for SCX). These methods were implemented on self‐packed 100 µm ID capillary columns at 500 nL/min to enhance sensitivity. To enable simultaneous analysis of acidic and basic species, we introduced a double‐barrel column configuration that integrates nanoSAX and nanoSCX in a noninterfering, parallel manner. This platform was applied to an *E. coli* cell lysate, identifying 301 unique proteoform masses (D‐score > 40) ranging from 10 to 150 kDa (with about 120> 50 kDa). Notably, the two modes were highly complementary, with only 10 overlapping species. The double‐barrel nanoflow IEC‐nMS platform provides a robust, sensitive, and high‐resolution strategy for native top‐down proteomics, enabling in‐depth study of complex proteomes.

## 1 Introduction

Native liquid chromatography (LC)‐ native mass spectrometry (nMS) methods have been attractive to nondenaturing protein analysis compared with traditional peptide‐centric approaches [1, 2, 3, 4], which employ water‐based solvents at native‐like conditions to maintain protein original states and preserve their labile noncovalent interactions [5, 6]. Among these native approaches, ion‐exchange chromatography (IEC) coupled with nMS stands out for its high resolving power in separating proteoforms based on their distinct surface charge distributions [7, 8, 9], and has been broadly applied in biopharmaceuticals, clinical studies, the food industry, etc.

**Statement of Significance of the Study**

1. Broad linear pH change profiles are achieved with volatile additives for SAX and SCX, precluding protein co‐elution and structure damage.

2. Nanoflow IEC‐nMS provides a nondenaturing condition for the analysis of intact proteins with enhanced sensitivity, benefiting for the low‐abundant proteoform identification.

3. Double‐barrel column configuration allows running of SAX and SCX in parallel and a noninterfering approach, resolving both acidic and basic proteoforms of complex samples in a high‐throughput and time‐saving way.

4. The developed platform provides a novel strategy for native top‐down proteomics and allows the comprehensive study of complex proteomes.

In IEC‐nMS, (i) the salt gradient elution approach employs high salt concentrations (typically up to 1 M) to elute proteins [10], which usually requires buffer exchange (or post‐column flow splitter) and harsh desolvation condition in electrospray ionization (ESI), resulting in the low sensitivity, protein denaturation, and ion suppression effects (where the analyte signal decreases as salt concentration increases) [11, 12]. In contrast, (ii) pH gradient methods (including the salt‐mediated pH gradient approach) exploit a low salt concentration, relying on pH changes to elute proteins, providing a better MS‐compatible environment [13, 14]. For instance, Yan et al. utilized a post‐column splitter to reduce the flow to 1 µL/min and developed an ammonium acetate (AmAc) based salt‐mediated pH gradient IEC‐MS method to characterize the charge heterogeneity of therapeutic monoclonal antibodies [15]. However, three key challenges still exist: (i) the applied flow splitter and required high amount of sample injection (e.g., 50 µg) represent the inadequate MS sensitivity, which is unfriendly to the low‐abundant protein detection. (ii) The limited selection of volatile salts, such as AmAc, ammonium formate (AmFo), and ammonium bicarbonate (AmBc), restricts both the usable buffer range and capacity, often resulting in abrupt pH change that can cause unexpected protein retention or co‐elution [16]. (iii) The single ion exchange mode (cation or anion) is usually insufficient to handle complicated biological samples, which contain a large quantity of basic (pI > 7) and acidic proteins (pI < 7) [17, 18]. Therefore, to ensure effective separations and detections for proteoform mixtures, it is critical to achieve a broad and linear pH gradient in both cation exchange (CEX) and anion exchange (AEX) approaches with high MS sensitivity.

An effective strategy to increase sensitivity is the use of low‐flow LC‐MS. Lower flow rates can reduce ion suppression effects by decreasing the relative amount of salt delivered to ESI, thereby enhancing MS sensitivity [11, 19]. Additionally, nanoflow‐ESI enables milder ionization conditions by eliminating the need for heated gas flow, helping to preserve labile structure of proteins (e.g., complexes) [20, 21]. However, the application of nanoflow IEC‐MS for complex protein analysis was not demonstrated until recently, when we introduced a nanoflow SCX‐nMS (500 nL/min) and applied this method to analyze an *E. coli* cell lysate sample, detecting both large and low‐abundance intact proteins with higher sensitivity than analytical flow IEC‐MS [22]. To achieve a linear pH response with volatile salts, three novel strategies have been proposed, including implementing new volatile additives, correcting nonlinear pH profiles, and applying salt‐assisted elution [9]. Nevertheless, the linear pH range for both AEX and CEX is still limited in less than 2 pH units currently, precluding the detailed separation of complex proteoforms.

Proteomics samples usually comprise proteins with a wide concentration range (dynamic range) and chemical diversity (broad pI distribution) [17, 23]. To retain and resolve all proteins within a single IEC analysis, different separation approaches have been applied: (i) tandem IEC, (ii) mixed‐bed IEC, and (iii) the development of 2DLC methods. (i) In 1986, Rassi and Maa proposed to use tandem columns to analyze the mixtures of basic and acidic proteins [24, 25]. However, the presence of two columns negatively affects the method's separation performance, as the gradient profile may be altered by the first column, and the second column adds significant band broadening to the analytes retained in the first column. (ii) Recently, mixed‐bed ion‐exchange columns have gained attention. Fischer et al. have developed an online mixed‐bed IEC to separate proteoforms from a complex human heart extract under native conditions using capillary flow [18]. Subsequently, this method was integrated into an SEC×IEC 2DLC workflow to analyze endogenous protein complexes [26]. However, preparing a homogeneous mixed‐bed column remains a challenge, and the columns are not commercially available. (iii) 2DLC is another valuable strategy to combine both AEX and CEX separations. Kumar et al. have successfully coupled CEX and AEX for the analysis of basic and acidic variants from recombinant monoclonal antibodies (mAbs) [27]. However, the coupling of anion and cation exchange was only demonstrated for the study of a single fraction from SCX to SAX (strong anion‐exchange chromatography). No further work explored the use of 2DLC to analyze complex samples, presumably due to the challenges of achieving complementary separations (e.g., what is retained under SAX conditions is not retained in SCX).

In this study, we describe a novel platform that uses nanoflow SCX and SAX directly coupled to native MS to characterize complex proteoform mixtures at the intact level. pH‐gradient‐based elution methods are employed in SAX (pH gradient) and SCX (salt‐mediated pH gradient) modes using MS‐compatible buffer systems. To achieve optimal resolution, the pH change over time is investigated using various types and amounts of volatile additives using analytical scale column. Nanoflow SAX and SCX are performed at 500 nL/min using self‐packed capillary columns (100 µm ID × 10 cm), exhibiting chromatographic behavior similar to that of the analytical flow. To simultaneously analyze acidic and basic proteins in complex samples, a novel double‐barrel configuration for the hyphenation of SAX and SCX is reported, enabling proteoform separation of the same sample by both methods using a single LC‐MS system. Finally, we use *E. coli* cell lysate, which contains a diversity of acidic and basic proteoforms, to demonstrate the capability of this strategy in analyzing complex, nondenatured samples at the intact level.

## 2 Materials and Methods

### 2.1 Chemicals and Materials

Bovine serum albumin (BSA, ≥ 96%), carbonic anhydrase from bovine erythrocytes (CA, ≥ 95%), myoglobin from equine skeletal muscle (Myo, 95%–100%), ribonuclease A from bovine pancreas (RNase‐A, ≥ 60%), trypsin inhibitor (TI) from glycine max (soybean), amyloglucosidase from aspergillus niger (Amy, 70 U/mg), and albumin from chicken egg white (Ova, ≥ 90%) were purchased from Sigma‐Aldrich (St. Louis, USA). Trastuzumab (Tra) was obtained from Roche (Grenzach‐Wyhlen, Germany). Cetuximab (Cet) was purchased from Merck (Amsterdam, The Netherlands). Pembrolizumab (Pem) was obtained from MSD (London, United Kingdom). Details of other materials and chemicals are reported in section S1.1. The summary of protein information is reported in Table S1. The instruments for analytical flow SCX and SAX‐UV/FLD can be found in section S1.2. Section S1.3 describes the instrument and materials for the nanoflow SCX and SAX‐nMS setup.

### 2.2 SCX and SAX‐UV/FLD

For SCX‐UV/FLD, a strong cation‐exchange column BioPro IEX SF (100 mm × 4.6 mm, 5 µm particle size, YMC, Japan) was utilized with a flow rate of 0.4 mL/min at room temperature. Mobile phases were optimized based on our previously published conditions [22]. To obtain a linear pH change in a wide range, eighteen types of mobile phases (Tables 1 and S2) were developed with a starting pH of 5.0. Their performances were assessed by separating a protein mixture (BSA, CA, RNase‐A, and Myo) and a mAbs mixture (Pem, Cet, and Tra) with a concentration of 1 mg/mL (dissolved in mobile phase A) using a gradient of 0‐0‐100‐100‐0‐0 %B in 0‐3‐33‐40‐40.1‐55 min. The injection volume was 5 µL, and UV absorbance was set at 280 nm. The excitation and emission wavelengths for the fluorescence detector (FLD) were 280 and 340 nm, respectively. The online pH meter recorded the pH profile during the entire analysis time.

**TABLE 1:** Summary of mobile phases used in SCX‐UV/FLD.

	Mobile phase A (mM)			Mobile phase B (mM)					
MP	AmAc	DFEA	pH	AmAc	DFEA	pH	Linear pH	n_c_	Peaks
**c1**	**20**	**0**	**5**	**250**	**0**	**8.5**	**5.0‐6.5**	**90**	**17**
c2	20	0	5	250	0	9.5	4.9‐5.8	111	4
c3	20	20	5	250	20	9.5	5.1‐7.5	92	16
c4	20	50	5	250	50	9.5	5.1‐7.2	83	15
c5	20	50	5	100	50	9.5	5.1‐7.6	75	16
c6	20	50	5	50	50	9.5	5.1‐7.8	72	18
c7	20	50	5	20	50	9.5	5.1‐8.0	69	15
c8	20	20	5	20	20	9.5	5.1‐7.9	55	13
c9	20	40	5	20	40	9.5	5.1‐7.9	36	17
c10^a^	20	40	5	20	40	9.5	5.2‐7.9	49	21
c11	20	40	5	140	40	8.5	5.1‐8.6	70	22

*Note*: MP: mobile phase; AmAc: ammonium acetate; DFEA: 2,2‐difluoroethylamine. The pH of mobile phase A is adjusted by acetic acid (AA) and the pH of mobile phase B is adjusted by ammonia hydroxide (AmHy).

^a^ this method uses a gradient correction method based on the pH profile. n_c_ indicates peak capacity. Extended results presented in supporting information section S2.

For SAX‐UV/FLD, a strong anion‐exchange column BioPro IEX QF (100 mm × 4.6 mm, 5 µm particle size, YMC, Japan) was used with a flow rate of 0.4 mL/min at room temperature. Ten different mobile phases with a starting pH of 7.5 were created (Table 2) and evaluated by analyzing acidic proteins (BSA, CA, TI, Ova, and Amy) with a concentration of 1 mg/mL (dissolved in mobile phase A). The other chromatographic conditions, including gradient methods, injection volume, and detector wavelength, were the same as the SCX‐UV/FLD.

**TABLE 2:** Summary of mobile phases used in SAX‐UV/FLD.

		Mobile phase A (mM)^a^			Mobile phase B (mM)									
MP	PBS	AmAc	AmFo	DFEA	PBS	NaCl	AmAc	AA	FA	DFEA	pH	Linear pH	n_c_	Peaks
a1	20				20	500					7.5	n.a.	39	23
a2		20					400				7.5	n.a.	42	26
a3		10	10					10	10		3.0	4.5‐3.0	64	20
a4		20	20					20	20		2.7	4.9‐2.7	64	23
**a5**		**30**	**30**					**30**	**30**		**2.6**	**5.0‐2.6**	**81**	**24**
a6		50	50					50	50		2.5	5.0‐2.5	84	16
a7		20	20	20				20	20	20	3.0	7.5‐3.0	68	37
**a8**		**20**					**140**				**4.5**	**n.a**.	**104**	**23**
a9		10	10					30	30		2.6	4.3‐2.5	73	21
a10		10	10					50	50		2.5	4.4‐2.6	80	23

*Note*: AmAc: ammonium acetate; AmFo: ammonium formate; AA: acetic acid; FA: formic acid; DFEA: 2,2‐difluoroethylamine. The pH of mobile phase A is adjusted by ammonia hydroxide. The pH of mobile phase B is adjusted by ammonia hydroxide (MPa01 and MPa02), FA (MPa07), or AA (MPa08). The pH of the rest of mobile phase B is not adjusted. n_c_ indicates peak capacity.

^a^ MPA was kept at pH 7.5.

### 2.3 Nanoflow SCX and SAX‐MS

Bulk materials of BioPro IEX SF and QF (5 µm, YMC) were exploited to prepare SCX and SAX capillary columns, respectively. These ion‐exchange resins were suspended in a packing solvent composed of 500 mM Na_2_SO_4_ and 50 mM PBS (pH 7.0) and ultrasonicated for 30 min. The acquired homogenous suspension was packed into an end‐sealed capillary column (100 µm ID × 15 cm) with a pump at the maximum limited pressure of particles (about 200 bar). The packing time lasted for an extra 10 min after finishing to stabilize and compact the column bed. The obtained capillary ion‐exchange columns were flushed with 20 mM AmAc solution over six hours to remove the nonvolatile salts and trimmed to 10 cm before use.

For nanoflow SCX‐MS, all the diameters of connection capillaries (e.g., the one between the autosampler and the column) were 20 µm to reduce the dead volume. The self‐packed capillary SCX column (100 µm ID × 10 cm) was run with a flow rate of 500 nL/min at room temperature. Three types of mobile phases (MPc11, MPc19, and MPc20, Tables 1 and S3) were assessed by separating the protein mixture (BSA, CA, RNase‐A, and Myo) and the mAbs mixture (Pem, Cet, and Tra) with a concentration of 0.5 mg/mL. A gradient of 1‐1‐99‐99‐1‐1 %B in 0‐2‐25‐30‐31‐45 min was used with 1 µL injection of these samples. For nanoflow SAX‐MS, the same dimensional capillary SAX column (100 µm ID × 10 cm) was evaluated at a flow rate of 500 nL/min without temperature control. Two types (pH gradient and salt‐mediated pH gradient) of mobile phases (MPa05 and MPa08, Table 2) were compared by analyzing 0.1 mg/mL of proteins (Oval, TI, Amy, and BSA) with 1 µL injection. The other nanoflow SAX‐MS parameters were identical to those of the nanoflow SCX‐MS.

The MS acquisition parameters for nanoflow SCX and SAX‐nMS were the scan mode of HRM, scan range 1000–8000 m/z, in‐source collision‐induced dissociation (isCID) values of 55 and 85 eV (only for mAbs), number of micro‐scans of 10, resolution of 17,500, automatic‐gain‐control (AGC) target of 3 × 10^6^, maximum injection time (IT) of 200 ms, spray voltage of 1.8 kV, transfer‐capillary temperature of 275°C, and S‐lens of 200 radio frequency.

### 2.4 Integration of Nanoflow SCX‐MS and SAX‐MS

To achieve the simultaneous analysis of acidic and basic proteins contained in a complex sample, three ways (series, parallel, and double‐barrel coupling) of integrating nanoflow SCX‐MS and SAX‐MS are compared. The series and parallel coupling ways are described in section S1.4. For double‐barrel coupling, a modified setup was developed based on previous literature [28]. Mobile phases of MPc01 (SCX) and MPa08 (SAX) are used with the same gradient of 1‐1‐99‐99‐1‐1 %B in 0‐2‐25‐30‐31‐45 min. The valve positions 1_2 (right) and 1_2 (left) are designated for SCX analysis, whereas 10_1 (right) and 10_1 (left) are reserved for SAX mode. The flow rate for these three coupling ways is 500 nL/min.

### 2.5 Data Analysis

Data from IEC‐UV/FLD were analyzed with the Agilent OpenLAB CDS Chemstation software. MS data were viewed with Thermo Xcalibur Qual Browser (v4.4) and Freestyle software (v1.7, Thermo Fisher Scientific). The UniDec v7.0.2 was used to perform deconvolution of mass spectra [29]. Total ion chromatogram (TIC) and extracted ion chromatogram (EIC) were smoothed using a 7‐point Gaussian filter. The pH linearity is calculated using the Pearson correlation coefficient and R^2^ correlation (OriginLab software) based on the recorded pH profiles. Peak capacities were calculated with the same approach as reported [30]. Protein mass features were obtained by deconvoluting our LC‐MS runs using UniDec using the D‐score metrics developed by Marty et al. [31]. The D‐score assesses data fitness, peak shape consistency, distribution smoothness, and peak symmetry. The accepted criteria are peak detection threshold > 0.1 and D‐score > 40. Proteforms were reported only if their masses differed by more than 2 Da. [32]. A complete list of all identified proteoform masses and their relative abundances for cell lysate is provided in the Supplementary Information.

## 3 Results and Discussion

Native IEC is a valuable method for characterizing proteoforms by preserving their native states and resolving them based on their surface charge distributions. However, direct IEC‐MS faces two key challenges: the limited choice of volatile salts often leads to nonlinear pH gradients and protein co‐elution. At the same time, a single‐column setup (cation or anion exchange) is inadequate to retain the broad distribution of proteoforms in biological samples due to their wide isoelectric point range.

To address these issues, we developed a nanoflow dual‐mode strong anion‐ and cation‐exchange (SAX/SCX) platform integrated into a double‐barrel column configuration. This platform enables coverage of a wide pH range (2.6 to 5 and 5 to 8.5) during gradient elution and employs milder desolvation conditions, resulting in high‐efficiency and high‐sensitivity LC‐MS separations. To develop this setup, we (i) optimized the type and concentrations of volatile additives for SCX and SAX using analytical flow IEC‐UV, (ii) transferred the method to nanoscale IEC‐MS, (iii) compared different approaches to integrate SCX and SAX methods, and finally (iv) applied this method for the analysis of an *E. coli* cell lysate.

### 3.1 Method Development and Characterization of Analytical Flow SCX and SAX‐FLD/UV

We initiated our work by performing IEC‐UV optimization at the analytical flow scale (0.4 mL/min). This approach allowed us to quickly screen buffer performance, specifically pH linearity and capacity, and to perform post‐column pH detection. A strategy increasingly employed to facilitate the direct coupling of IEC and MS is the use of a pH gradient with volatile additives. However, existing methods in the literature are often developed for targeted protein studies (e.g., monoclonal antibodies) and are insufficient for analyzing complex protein mixtures. To the best of our knowledge, the reported linear pH range for SCX and SAX using volatile buffers is 5.0‐6.5 and 4.0‐2.8, respectively [22, 33]. Therefore, our investigation began with the development of SCX and SAX methods capable of spanning a wider pH range. We first focused on SCX and then extended our efforts to SAX. These methods were evaluated by monitoring post‐column pH gradient profiles and assessing chromatographic separations using reference proteins with different pI values (Table S1).

**FIGURE 1:**
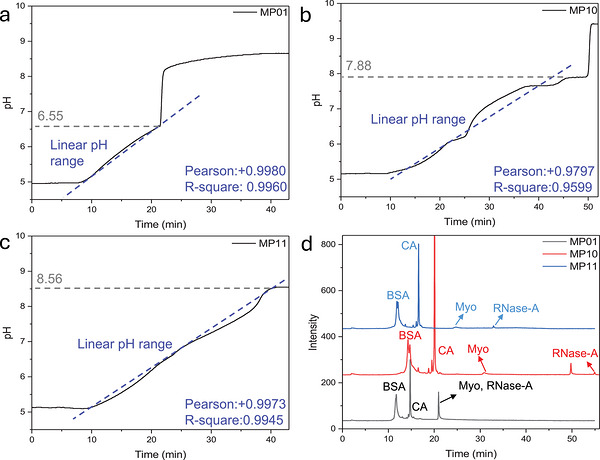
(a‐c) The pH profiles of different mobile phases (MPc01, MPc10, and MPc11) in SCX‐UV/FLD. (d) Separation of a protein mixture (BSA, CA, Myo, and RNase‐A) using different mobile phases (MPc01, MPc10, and MPc11) in SCX‐UV/FLD.

Our starting point in the SCX optimization was a method we recently described using 20 mM AmAc (pH 5) and 250 mM AmAc (pH 8.5) (MPc01, Figure 1a) [22]. We investigated a range of mobile phase composition parameters, including the effect of mobile phase concentration (20‐250 mM), the use of different buffer additives (AmAc, DFEA), pH range (5‐9.5), and pH gradient correction. The scope of our optimization was to develop a method capable of generating a linear pH profile over a broad range (e.g., 5 to 9.5) and/or maintaining a relatively low ionic concentration (e.g., below 300 mM), thereby improving the separation of basic proteoforms under MS‐compatible conditions (MPc02‐MPc11). A complete overview of the mobile phase conditions tested is provided in Table 1.

The performance of methods is assessed by the pH change over time, referred to as pH linearity. To characterize it, we used the Pearson correlation coefficient and the R^2^ value from a linear fit of the pH change over time. We observed that extending the end pH of mobile phases from 8.5 to 9.5 (MPc02) resulted in a sharp pH increase from approximately 6 to 9 within 0.9 min (Figure S1a), indicating the poor buffer capacity of AmAc in this range. Therefore, we tested other MS‐compatible additives to extend this range. Davis et al. found that 2,2‐difluoroethylamine (DFEA) can offer buffering capacity where the AmAc falls short [34]. We incorporated DFEA into mobile phases and investigated the influence of DFEA and AmAc concentrations on the pH change (MPc03‐MPc09). Figure S1b–S1h showed the capacity of DFEA to buffer pH between 6 and 8, with an increase in pH linearity (Pearson/R^2^) from around 0.84/0.70 to 0.97/0.94.

Additionally, a persistent challenge is the lack of buffer capacity at high pH (8–9.5), leading to sudden pH changes within seconds. This issue was also observed with other volatile additives, such as ammonium bicarbonate, 2‐fluoroethylamine hydrochloride, and methylamine, which exhibit weak pH control in this critical high‐pH range (MPc12‐MPc18), as shown in Figure S2 and Table S2. To address this, we adopted a strategy proposed by Fekete et al., which uses an inverse function of the pH response to empirically correct a nonlinear pH gradient [35]. Applying this to the gradient of MPc09, we created a corrected gradient of MPc10 (Figure S3). This additional approach further improved the pH linearity (Pearson/R^2^) from 0.96/0.92 to 0.98/0.96 (MPc10, Figure 1b). However, buffering the pH above 8 remained challenging even when applying gradient correction approaches, as it fell outside the correction range. Therefore, we opted to narrow the final pH to 8.5 (MPc11). This allowed us to obtain a highly linear pH range from 5 to 8.5, with Pearson/R^2^ values of 1.00/0.99 (Figure 1c) using no gradient programming correction and therefore a simpler method program.

To evaluate the effect on chromatographic performance, we tested the separation of mixtures of reference proteins (BSA with pI around 5.0, CA with pI 6.6, Myo with pI 7.0, and RNase‐A with pI 9.6) and mAbs (Pem with pI 7.6, Cet with 8.8, and Tra with pI 9.1). The separation performance was assessed based on peak capacity and the number of resolved peaks. When a rapid pH change occurs, proteins and mAbs can co‐elute together or have reduced resolution (Figures S4 and S5). Methods with improved pH linearity generally resulted in better separations, demonstrating the importance of pH control in IEC analysis with pH‐based gradient modes. MPc11 showed the best performance, with a peak capacity of 70 and the highest number of protein peaks (22 when summing all peaks in protein and mAb mixtures; Table 1). While other volatile buffer combinations (MPc12‐c18, incorporating DFEA and MA) were tested, they provided no significant improvement. Finally, MPc01 can also be considered a high‐performance mobile phase method, given its good separation performance (peak capacity of 90 and 17 peaks resolved). Here, the high concentration of salts present in MPB allows for protein elution in the linear pH change window [9]. We therefore selected the MPc01 and MPc11 methods, based on their excellent proteoform separation performance in analytical flow experiments, for nanoflow SCX‐MS.

**FIGURE 2:**
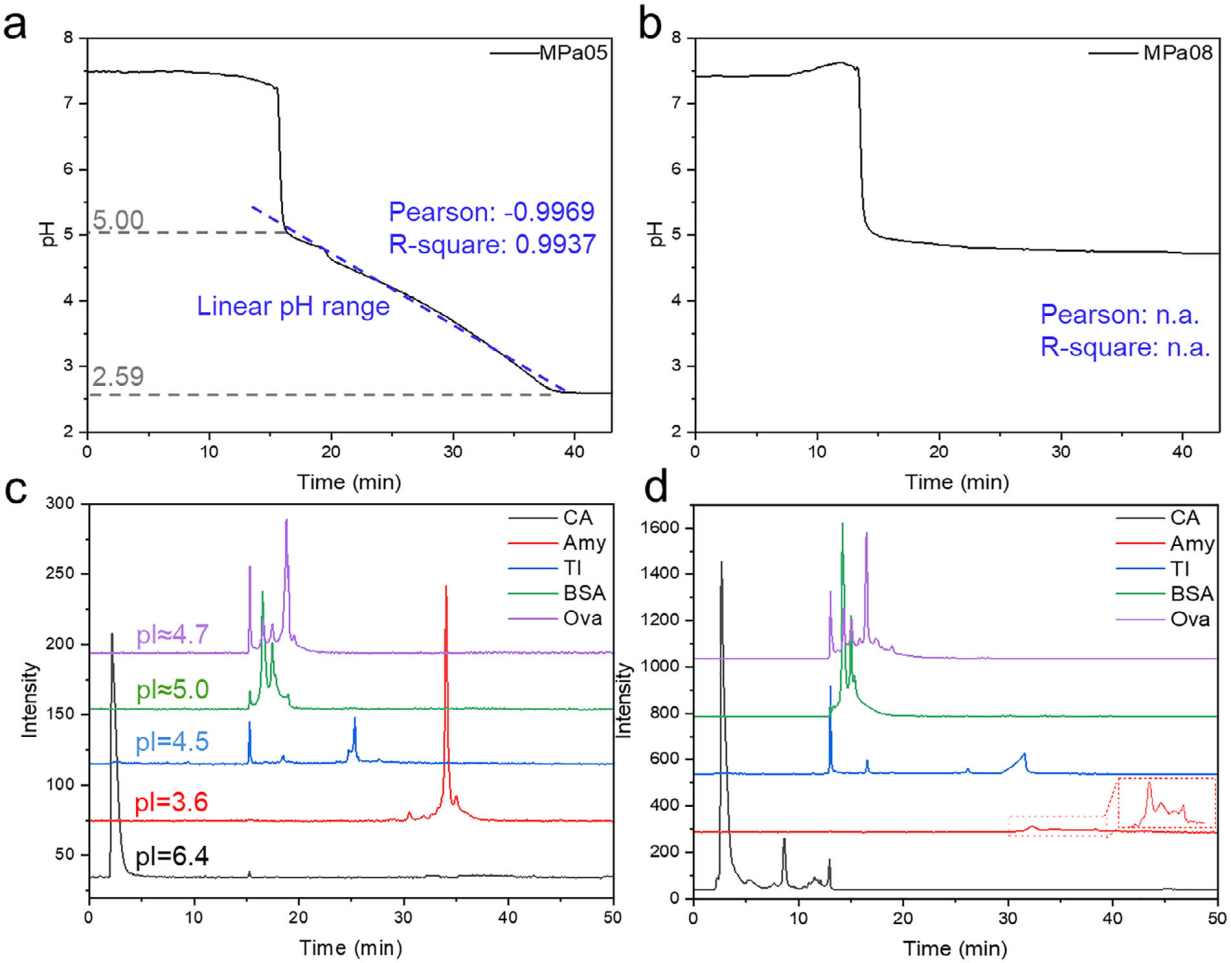
The pH profiles of MPa05 (a) and MPa08 (b) in SAX‐UV/FLD. Performances of the MPa05 (c) and MPa08 (d) in separating charge variants of various acidic proteins (CA, Amy, TI, BSA, and Ova) using SAX‐UV/FLD.

Next, we explored diverse SAX‐UV/FLD methods in acidic proteoform separation. We first assessed the performance of the salt‐gradient method (MPa01) using nonvolatile (NaCl) salts at pH 7.5 (Figure S6). We then incorporated the volatile AmAc/AA and AmFo/FA buffers into the mobile phases (MPa02‐MPa06) to create a salt and pH gradient starting at pH 7.5. In pH gradients, the different concentrations of these buffers had a slight influence on pH linearity (Figure S7), but MPa05 provided the best pH profile, exhibiting a linear change between 5.0 and 2.6 with Pearson/R^2^ values of approximately 1.0/1.0 (Figure 2a). The addition of DFEA (MPa07) successfully offset the lack of buffer capacity between pH 7.5 and 5, but at the cost of reduced pH linearity, which dropped to 0.94/0.88 (Figure S7d). We also investigated three salt‐mediated pH gradient methods (MPa08‐MPa10), all of which exhibited lower pH linearity (Figures 2b and S8).

Similarly to what has been described for the SCX methods, the SAX methods were tested for their separation performance using reference proteins. Five acidic proteins (CA, Amy, TI, BSA, and Ova, Table S1) of varying sizes (20 to 66 kDa) and pI values (3.6 to 6.4) are used to assess the separation performance of SAX‐UV/FLD using MPa01‐MPa10 (Table 2 and Figures S9–S11). These model proteins are chosen for their low pI and distribution in proteoforms. In particular, Amy and Ova are heterogeneous proteins that present glycosylation, acetylation, and phosphorylation modifications [36].

When comparing salt gradient mode, the AmAc‐based method (MPa02) showed better resolution for charge variants of CA, Ova, BSA, and TI compared with nonvolatile additives (MPa01, Figure S9) as demonstrated by the higher number of peaks observed (26 vs. 23, Table 2). However, MPa02 was insufficient to elute the more acidic Amy proteoforms (pI = 3.6). In comparison to the salt‐gradient method of MPa02, the pH gradient methods (MPa03‐MPa07) with final pH values between 2.5 and 3 yielded a peak capacity above 64 with respect to about 40 for MPa02 (Table 2 and Figure S10). MPa05 (Figure 2c) demonstrated higher separation performance (peak capacity 81), benefiting from better pH linearity over a broader range (detecting 24 protein peaks). Although the salt‐mediated pH gradient method of MPa08 presents a flat pH profile, it achieves good separation with high capacity (peak capacity of 104, 23 peaks detected; Figure 2d and Table 2). Based on our results, we selected methods MPa05 and MPa08 to perform the proteoform separation in the nanoflow SAX‐MS measurements.

### 3.2 Performance Evaluation of Nanoflow SCX and SAX‐nMS

The developed methods from the analytical flow (0.4 mL/min) SAX‐ and SCX‐UV/FLD were adopted to nanoflow (500 nL/min) SAX‐ and SCX‐nMS using packed capillary columns (100 µm × 10 cm, see experimental section for details). We began the nanoflow SCX‐nMS optimization by testing MPc11 (containing DFEA) in the analysis of a three‐protein mixture (BSA, CA, and RNase‐A) (Figure S12a). This method achieved good separation performance, yielding an average peak width at half height (w_h_) of 0.3 min.

**FIGURE 3:**
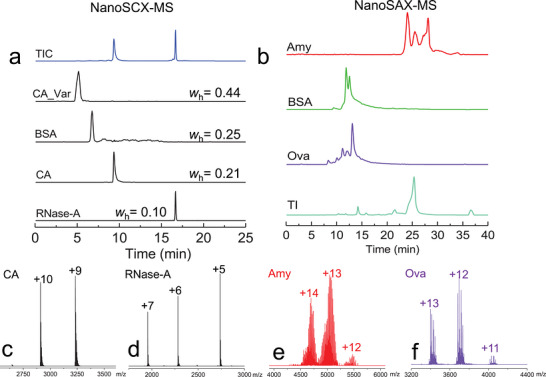
(a) Protein mixture (CA, BSA, and RNase‐A) analysis using nanoflow SCX‐MS with a mobile phase of MPc01. The m/z values used to extract EICs are shown in Table S5. (b) Proteoform separations of Amy, BSA, Ova, and TI by nanoflow SAX‐MS with a mobile phase of MPa08. (c‐f) Average mass spectra of representative proteins. CA (c, average time: 9.3‐9.6 min) and RNase‐A (d, average time: 16.6‐16.7 min) are obtained from nanoflow SCX‐nMS. Amy (e, average time: 23.5‐24.0 min) and Ova (f, average time: 12.8‐13.5 min) are obtained from nanoflow SAX‐nMS.

We then compared MPc11 with two previously reported mobile phases, MPc01 and MPc19 (composed of AmAc and/or ammonium bicarbonate; Table S3) [15, 22]. While MPc01 and MPc19 provided similar chromatographic separation efficiency with w_h_ of 0.25 and 0.30 min, respectively (Figures 3a and S12c), their sensitivity profiles varied (Table S4). MPc01 (AmAc only) exhibited the highest sensitivity (4.84E7). In contrast, other volatile additives, although present at lower molar concentrations than AmAc, significantly suppressed the signal: MPc11 (DFEA) produced a 55% reduction, and MPc19 (ammonium bicarbonate) an 82% reduction.

All tested mobile phases can be considered nondenaturing because they are water‐based (no co‐solvent added) and have a pH close to 7 (ranging from 4.5 to 9.5). The protein mass spectra showed a reduced charge‐state distribution, typical of native MS analysis (Figures 3 and S12). In particular, however, MPc01 produced the most native‐like MS conditions, with the most abundant charge state of +5 for RNase‐A (Figure 3d), which is lower than the +6 observed with MPc11 (Figure S12b) and the +7 with MPc1920 (Figure S12d).

We concluded that the addition of volatile additives DFEA and ammonium bicarbonate, although it improves IEC pH linearity and decreases the ionic concentration in the mobile phase, decreases LC‐MS performance. As the aim of the investigation was to achieve sensitive method with high separation performance, we selected MPc01 for subsequent measurements.

Next, to select the conditions for nanoflow SAX‐nMS, we compared methods based on MPa05 and MPa08 for LC‐MS separation of charge variants of four acidic proteins: Amy, BSA, Ova, and TI. MPa05 could separate numerous proteoforms at the intact level. Four peaks representing different variants and aggregates were observed for BSA (Figure S13c,d), and nine peaks corresponding to various modifications for Ova were obtained (Figure S14a,b). Several unknown species were also detected in the TI sample (Figure S14c,d), possibly due to protein hydrolysis. However, complex and unclear mass spectra of Amy (Figure S13a,b) were observed under these chromatographic conditions, likely due to different levels of adduct formation (e.g., Na^+^) that result in many complex MS signals or incomplete desolvation.

Under the same nanoflow SAX‐nMS conditions (Figure 3b), MPa08 achieved a significantly more powerful separation of Amy (Figure S15a,b), resolving 12 proteoforms with clear yet complex charge state envelopes, which included many glycovariants in the 4400–5500 m/z range. This finding has not been previously reported. The other proteins (BSA, Ova, and TI) analyzed with MPa08 yielded results similar to those obtained with MPa05 (Figures S15 and S16), but with significantly clearer mass spectra (i.e., reduced adduct formation). We believe that the more acidic conditions of MPa05 likely had adverse effects on the protein ionization compared to the near‐neutral pH of MPa08. In addition, AmFo, as a chaotropic salt, may disrupt hydrophobic interactions in proteins relative to AmAc, leading to structural damage [37]. Therefore, MPa08 was selected as the mobile phase for nanoflow SAX‐nMS and used in all subsequent experiments.

Finally, nanoflow SCX (MPc01) and SAX (MPa08)‐nMS successfully resolved charge variants of reference proteins with high sensitivity and selectivity. Both ion‐exchange modes (SCX and SAX) produced clean, simple native mass spectra with low charge states and narrow charge‐state envelopes, thereby enabling characterization of complex proteoforms by native LC‐MS. Notably, the high pH linearity in pH‐based elution strategies increases the likelihood of separating proteoforms in complex protein mixtures with broad pI distributions. To maximize sensitivity and minimize pH‐induced protein denaturation, AmAc‐only (MPc01 SCX and MPa08 SAX) systems are recommended. However, for resolving complex mixtures with overlapping pIs, the DFEA‐based (e.g., SCX MPc11) and lower‐pH SAX (e.g., SAX MPa05) mobile phase gradients offer better separation but reduce signal intensity.

For maximum sensitivity, AmAc‐only systems are recommended; however, for resolving complex mixtures with overlapping pIs, the DFEA‐based linear gradient provides superior separation at the cost of some signal intensity.

### 3.3 Combined Nanoflow SCX‐ and SAX‐nMS and Its Application to the Analysis of an *E. coli* Cell Lysate

To characterize complex biological samples that contain a variety of acidic and basic proteins, we describe and test, for the first time, the integration of nanoflow SCX and SAX separations in a single system using three coupling configurations: series, parallel, and double‐barrel. Unlike two‐dimensional liquid chromatography (2DLC) approaches, which transfer fractions from a first column to a second, our combined method analyzes sample components that were not retained on the first column in the complementary IEC mode.

**FIGURE 4:**
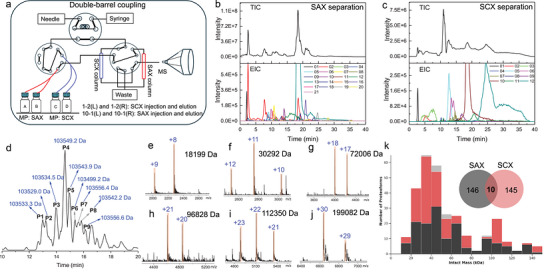
(a) Workflow of the double‐barrel coupling. (b) Results of *E. coli* cell lysate analyzed by nanoflow SAX‐nMS and (c) SCX‐nMS using the double‐barrel coupling (mass range: 2000–8000). The m/z used to obtain the EIC in figure b and c are reported in Table S6. (d) An example of the high‐MW proteoform separation using SCX mode in the double‐barrel coupling. (e‐j) Average mass spectra of representative species observed in the cell lysates (the average time range of them is listed in Table S7). (k) A histogram describing the distribution of the deconvoluted masses (D‐score >40) identified in the analysis of the *E. coli* cell lysate using the double‐barrel coupling. Duplicate masses identified in both SAX and SCX runs (within 1 Da) were consolidated to prevent overestimation of proteoforms.

In both series and parallel coupling, the sample is injected on the first column (SCX for series and SAX in the case of parallel), and the unretained sample is collected on the second column. For series coupling (Figure S17a), the SCX and SAX columns are directly linked via a single switching valve that switches between IEC elution modes. In parallel coupling (Figure S17b), the SCX and SAX columns run separately by using two switching valves. Both coupling methods require a single starting mobile phase, which runs over the SAX and SCX columns. The starting mobile phase must be a compromise between the starting conditions of the two modes. Overall, this compromise separation performance as the initial pH environment influences the IEC separations. In contrast, the double‐barrel coupling (Figure 4a) requires two separate injections of the same samples and allow for independent operation and mobile phase choice for SCX and SAX methods. This noninteractive approach avoids pH discrepancies caused by sample loading and significantly improves analysis efficiency by allowing one column to pre‐equilibrate while the other is in use.

The performance of these three coupling methods was evaluated and compared using a set of reference proteins, including BSA and Amy (Figure S18). In the series coupling method, the analysis of BSA resulted in broad peaks distributed across both the SAX separation area (0‐36 min) and the SCX area (36‐90 min) (Figure S18a), suggesting the presence of undesirable secondary interactions in the SCX method (second separation). Similarly, parallel coupling failed to resolve the BSA variants, producing a single broad peak that spanned the 20–40 min range (Figure S18b). In contrast, the double‐barrel column configuration successfully separated BSA charge variants into narrow peaks, with performance comparable to that of a single nanoflow SAX‐nMS run (Figure S18c). Finally, when analyzing Amy (Figures S18d–f), the double‐barrel coupling also demonstrated superior selectivity compared to the other coupling methods. This highlights its potential to maintain the separation efficiency of a single SAX or SCX analysis while processing more complex samples. The double‐barrel coupling was therefore selected for the analysis of a cell lysate.

To explore the performance of the double‐barrel coupling in analyzing complex samples, we used an *E. coli* cell lysate containing a broad mixture of acidic and basic proteins (with a concentration of 4.86 mg/mL via Bradford assay). The SDS‐PAGE results confirmed a wide range of protein molecular weights in this sample (Figure S19). 1 µL of the cell lysate was injected into the SAX and SCX modes. The total run time of the method (combined SAX, SCX, and washes) was 90 min. A low‐mass polymer contaminant (MW 1000 to 2000 m/z), likely a nonionic surfactant from the lysis buffer, was detected but eluted early and did not interfere with the main protein separation window. Carry‐over effects were observed in both SAX and SCX modes, requiring three blank runs (injecting mobile phase A, two SCX and one SAX runs) between injections (SCX carryover of 10% was observed in the first blank for the species shown in EIC09 of Figure 4, which reduced to below 2% in the second blank).

Both separation modes revealed multiple proteoform distributions in the direct analysis of the *E. coli* sample. The SAX run, designed for acidic species (pI from high to low), yielded a complex TIC spanning approximately 2 to 36 min (Figure 4b). Similarly, the SCX run, separating basic species (pI low to high), eluted proteins between 2 and 40 min (Figure 4c). To further analyze the method's performance, we extracted 12 ion chromatograms of protein features spanning a broad molecular weight range (15‐150 kDa). Overall, good separation performance was observed for both methods, with peak capacities (based on a 23 min gradient) of 21 for SAX and 12 for SCX (3 replicate runs reported similar results in terms of retention time, <2% relative time difference, and peak capacity within 5%). In Figure 4e–j (deconvoluted data in Figure S21), we show 8 representative protein features. The resulting MS spectra consistently showed low charge states and narrow charge‐state envelopes, suggesting that the native structures of these diverse intact proteins were successfully preserved. Notably, in the SCX separation, we resolved approximately nine distinct proteoforms (eluting at different times) of a single 103 kDa protein, despite their minor mass differences (Figures 4d and S20).

Finally, we collected the proteoform results obtained by deconvolving the two separations in the histogram shown in Figure 4k. 301 species with a D‐score > 40 [32] with molecular weights from 10 to 150 kDa were detected by the double‐barrel coupling. We observed a wide dynamic range of protein signal intensity, spanning from 10^4^ to 10^7^ (nondeconvolved signal). Notably, the species represent the total after removing duplicate features identified by both SCX and SAX runs (within a window of 1 Da). The SAX contributed 146, and the SCX contributed 145, with only 10 overlapping (probably because both modes cover the same pH range of 5 to 7.5), underscoring the complementary nature of the two modes (Venn diagram, Figure 4k). Furthermore, the detected masses primarily represent proteoforms exceeding 30 kDa (above 80%), with a significant portion (over 10%) exceeding 100 kDa. In addition, the number of detected species increased to around 550 when the D‐score threshold was set to >20 (Figure S22).

## 4 Conclusions

In this study, we developed an online nanoflow dual IEC‐nMS platform integrating SAX and SCX to achieve a method with relatively high sensitivity that allows for the study of both acidic and basic proteins in a complex sample with a broad range of pI values and MWs. To preclude the rapid pH change‐induced protein co‐elution, increase the selectivity for proteoforms, and realize the direct coupling with native MS, we optimized the volatile additives‐based mobile phases of SAX and SCX to obtain a wide linear pH change zone from 2.6 to 5 (SAX) and 5 to 8.5 (SCX) for the pH‐dominated gradient method in analytical flow IEC‐UV/FLD. Then we downsized the separation to nanoscale SAX‐ and SCX‐nMS method using capillary columns (100 µm ID × 10 cm) at a flow rate of 500 nL/min. Finally, we combined nanoflow SAX and SCX in a double‐barrel method that enables running of two IEC modes in a noninterfering and noninteractive manner, thereby maintaining their separation power while approaching that of single SAX/SCX‐nMS analysis. Using this method, we performed an in‐depth analysis of E. coli cell lysate, separating acidic proteins (pI < 7.5) by SAX and basic proteins (pI> 5) by SCX within 90 min. The combined method allowed the detection of 471 unique masses (D‐score > 40) with the MW from 10 to 150 kDa. This study presents a technological advancement in IEC‐nMS for characterizing complex samples, offering opportunities for native top‐down proteomics and clinical research.

## Funding

The author has nothing to report.

## Conflicts of Interest

The authors declare no conflicts of interest.

## Supporting information




**Supporting File 1**: pmic70134‐sup‐0001‐SuppMat.docx.

**Supporting File 2**: pmic70134‐sup‐0002‐table.xlsx.

## Data Availability

Data is available on: ftp://massive‐ftp.ucsd.edu/v11/MSV000099515/.
