# Supplementary material for: Parallel Analysis of Acidic and Basic Proteoforms in Cell Lysates via Native Cation and Anion Exchange Chromatography—Native Mass Spectrometry
Source: Proteomics. 2026 Apr 21;26(7):158–68. doi: 10.1002/pmic.70134 (PMC13327707; doi:10.1002/pmic.70134)
Supplement: Supplementary file 1 — Supporting File 1: pmic70134‐sup‐0001‐SuppMat.docx. [file PMIC-26--s001.docx]

**Supplementary Information**

**Native Cation and Anion Exchange Chromatography—Mass Spectrometry for the Parallel Analysis of Acidic and Basic Proteoforms in Cell Lysates**

Ziran Zhai^†‡*^, Hafsa Zakri^†‡^, Matteo Damian^†^, Francesco G. Mutti^†^, Garry Corthals^†‡^, Andrea F.G. Gargano^†‡*^

^†^Analytical Chemistry Group and Biocatalysis Group, Van’t Hoff Institute for Molecular Sciences (HIMS), University of Amsterdam, Science Park 904, 1098 XH Amsterdam, The Netherlands.

^‡^Centre for Analytical Sciences Amsterdam, Van’t Hoff Institute for Molecular Sciences (HIMS), University of Amsterdam, Science Park 904, 1098 XH Amsterdam, The Netherlands.

*Corresponding author: Andrea F.G. Gargano; Ziran Zhai

E-mail: [a.gargano@uva.nl](mailto:a.gargano@uva.nl), [z.zhai@uva.nl](mailto:z.zhai@uva.nl)

Table of Contents

[S1. Experimental section 3](#_Toc220350247)

[S1.1 Chemicals and materials 3](#_Toc220350248)

[S1.2 Instruments of SAX and SCX-UV/FLD 3](#_Toc220350249)

[S1.3 Instrument of nanoflow SAX and SCX-nMS 3](#_Toc220350250)

[S1.4 Series and parallel coupling methods 4](#_Toc220350251)

[S2. Supplemental figures 5](#_Toc220350252)

[S2.1 The pH profile and separation performance of analytical flow SCX-UV/FLD 5](#_Toc220350253)

[S2.2 The pH profile and separation performance of analytical flow SAX-UV/FLD 8](#_Toc220350254)

[S2.3 Performance evaluation of nanoflow SAX and SCX-nMS 11](#_Toc220350255)

[S2.4 Integration of nanoflow SAX and SCX-nMS and performance evaluation 15](#_Toc220350256)

[S3. Supplemental tables 18](#_Toc220350257)

[S4. CRediT authorship contribution statement 20](#_Toc220350258)

[S5. References 20](#_Toc220350259)

# S1. Experimental section

## S1.1 Chemicals and materials

Ammonium acetate (AmAc, ≥ 98%), ammonium formate (AmFo, ≥ 99.0%), ammonium bicarbonate (AmBc, ≥ 99.5%), ammonium carbonate (AmCa), ammonia solution (25%), sodium chloride (NaCl, ≥ 99.5%), sodium phosphate dibasic (≥ 98.5%), and sodium phosphate monobasic (≥ 99.0%) were purchased from Sigma-Aldrich (Steinheim, Germany). Acetic acid (100%) and sodium sulfate (≥ 99%) was obtained from Merck (Darmstadt, Germany). 2,2-difluoroethylamine (DFEA, 97%), 2-fluoroethylamine hydrochloride (MFEA, 95%), and methylamine (MA, 40% w/w) were obtained from abcr GmbH (Karlsruhe, Germany). Formic acid (FA, 99%) was purchased from Biosolve chimie (Valkenswaard, The Netherlands). The ultrapure water (18.2 MΩcm) was produced by a Milli-Q purification system (Millipore, Bedford, MA, USA). Fused-silica capillaries (0.10 mm I.D., 0.36 mm O.D.) were purchased from CM Scientific (Silsden, UK). The frit kit (including formamide, Kasil-1, and Kasil-1624) was purchased from Next Advance (Troy, NY, USA). The information of proteins is summarized in Table S1. The *E. coli* cell lysate was prepared according to the procedure we described earlier and used to evaluate the performance of the proposed configuration.^1^ In brief, the cell lysate was freeze-dried and reconstituted with 1 mL of 20 mM AmAc. Then the sample was centrifuged with spin filters (3 kDa cutoff MW) at 5 °C and 12,000 rpm. Before the analysis, the obtained sample (around 100 µL) was diluted 10 times using the mobile phase A of SAX and SCX. The observed precipitants during the diluting process need to be removed using centrifugation at 5 °C and 12,000 rpm.

## S1.2 Instruments of SAX and SCX-UV/FLD

SAX and SCX-UV/FLD measurements were performed at analytical flow scale on an Agilent 1100 series Infinity HPLC system (Agilent, Waldbronn, Germany), which included a binary pump (G1312A), a 1260 high-performance degasser (G4225A), an autosampler (G1329A), a column compartment (G1316A), a multi-wavelength detector (MWD, G1365B), and a fluorescence detector (FLD, G1321A). The Agilent OpenLAB CDS Chemstation Edition (version 3.2) software was employed to control the HPLC system. A monitor pH/C-900 (Cytiva, USA) and a 1200 infinity universal interface box II (Agilent Technologies) were used to achieve the online pH measurements.

## S1.3 Instrument of nanoflow SAX and SCX-nMS

Nanoflow SAX and SCX were both performed on an UltiMate RSLCnano system (Thermo Fisher Scientific, Breda, The Netherlands) equipped with a high-pressure pump and a loading pump (NCS-3500RS), a binary nano/capillary pump (NCP-3200RS), a thermostatted column compartment (equipped with two 10-port, two-position valves) and an autosampler. 1 µL of injection loop was used and the autosampler was kept at 5 °C during the analysis. A Q Exactive-Plus Biopharma high-resolution mass spectrometer (Thermo Fisher Scientific, Bremen, Germany) was employed. Nano-spray ionization was realized with a nano-spray-flex-series ion-source (Thermo Fisher Scientific), a Simple Link UNO (1/32, Fossiliontech, Albacete, Spain), and a LOTUS nano-emitter (75 mm length × 20 µm I.D., Fossiliontech) with hydrophobic coatings.

## S1.4 Series and parallel coupling methods

For the series coupling, the mobile phases of MP05 (SAX) and MP11 (SCX) are employed with two gradients of 1-1-99-99-1-1 %B in 0-3-23-33-34-110 minutes (SAX) and 1-1-99-99-1-1 %B in 0-57-77-87-88-110 minutes (SCX). The valve switched at 0-36-90 minutes to the position of 1_2-10_1-1_2 (both capillary SCX and SAX columns are online during this period). For the parallel coupling, two gradients of 1-1-99-99-1-1 %B in 0-10-30-65-66-90 minutes (SCX) and 1-1-99-99-1-1 %B in 0-43-63-73-74-90 minutes (SAX) are run using the same mobile phases with the series coupling. The switching valves are run at 0-8-41-90 minutes to 1_2-1_2-10_1-1_2 (left valve) and 1_2-10_1-10_1-1_2 (right valve).

# S2. Supplemental figures

## S2.1 The pH profile and separation performance of analytical flow SCX-UV/FLD

**Figure S1**. Recorded pH profiles of mobile phases in SCX-UV/FLD. MP02 (a), MP03 (b), MP04 (c), MP05 (d), MP06 (e), MP07 (f), MP08 (g), and MP09 (h). Details of mobile phases are shown in Table 1.

**Figure S2**. Recorded pH profiles of extra methods in SCX-UV/FLD. MP12 (a), MP13 (b), MP14 (c), MP15 (d), MP16 (e), MP17 (f), and MP18 (g). Details of mobile phases are shown in Table S2.

**Figure S3**. Correction of the original gradient program. The pH profile of MP09 is converted to a function of gradient time, which rescales the pH signal in a range between 0 and 1. Then the inverse function is created based on the original function. The original (MP09) and corrected gradient program (MP10) are shown in the right two tables.

**Figure S4**. Separations of protein mixture using diverse mobile phases in SCX-UV/FLD. (a) MP02-MP04; (b) MP05-MP07; (c) MP08-MP09. CA: carbonic anhydrase; BSA: Bovine serum albumin; Myo: myoglobin; RNase-A: ribonuclease a.

**Figure S5**. Separations of the mAb mixture using diverse mobile phases in SCX-UV/FLD. (a) MP01, MP10, and MP11; (B) MP02-MP04; (C) MP05-MP07; (D) MP08-MP09. Pem: Pembrolizumab; Cet: Cetuximab; Tra: Trastuzumab.

## S2.2 The pH profile and separation performance of analytical flow SAX-UV/FLD

**Figure S6**. Recorded pH profiles of salt-gradient methods in SAX-UV/FLD. (a) MP01; (b) MP02. Details of mobile phases are shown in Table 2.

**Figure S7**. Recorded pH profiles of pH-gradient methods in SAX-UV/FLD. (a) MP03; (b) MP04; (c) MP06; (d) MP07. Details of mobile phases are shown in Table 2.

**Figure S8**. Recorded pH profiles of salt-mediated pH gradient methods in SAX-UV/FLD. (a) MP08; (b) MP09; (c) MP10. Details of mobile phases are shown in Table 2.

**Figure S9**. Separations of acidic proteins using salt-gradient methods in SAX-UV/FLD. (a) MP01; (b) MP02. CA: carbonic anhydrase; Amy: amyloglucosidase; TI: trypsin inhibitor; BSA: bovine serum albumin; Ova: ovalbumin.

**Figure S10**. Separations of acidic proteins using pH-gradient methods in SAX-UV/FLD. (a) MP03; (b) MP04; (c) MP06; (d) MP07. CA: carbonic anhydrase; Amy: amyloglucosidase; TI: trypsin inhibitor; BSA: bovine serum albumin; Ova: ovalbumin.

**Figure S11**. Separations of acidic proteins using salt-mediated pH gradient methods in SAX-UV/FLD. (a) MP08; (b) MP09; (c) MP10. CA: carbonic anhydrase; Amy: amyloglucosidase; TI: trypsin inhibitor; BSA: bovine serum albumin; Ova: ovalbumin.

## S2.3 Performance evaluation of nanoflow SAX and SCX-nMS

**Figure S12**. MS results of protein mixtures obtained from nanoSCX-nMS. Different mobile phases are compared to select the best condition. (a-b) MP11; (c-d) MP20. The m/z values used to extract EICs are shown in Table S4.

**Figure S13**. MS results of Amy and BSA using MP05 in nanoSAX-nMS. (a) TIC and EIC of Amy; (b) Mass spectra of Amy; (c) TIC and EIC of BSA; (d) Mass spectra of BSA.

**Figure S14**. MS results of Ova and TI using MP05 in nanoSAX-nMS. (a) TIC and EIC of Ova; (b) Mass spectra of Ova; (c) TIC and EIC of TI; (d) Mass spectra of TI.

**Figure S15**. MS results of Amy and BSA using MP08 in nanoSAX-nMS. (a) TIC and EIC of Amy; (b) Mass spectra of Amy; (c) TIC and EIC of BSA; (d) Mass spectra of BSA.

**Figure S16**. MS results of Ova and TI using MP08 in nanoSAX-nMS. (a) TIC and EIC of Ova; (b) Mass spectra of Ova; (c) TIC and EIC of TI; (d) Mass spectra of TI.

## S2.4 Integration of nanoflow SAX and SCX-nMS and performance evaluation

**Figure S17**. Workflow of series coupling (a) and parallel coupling (b).

**Figure S18**. Comparison of three coupling methods. TIC and EIC of BSA (a-c) and Amy (d-f) obtained from series, parallel, and double-barrel coupling methods, respectively. The red color represents the SAX separation mode, and the blue color means the SCX separation mode.

**Figure S19**. SDS-PAGE results of the *E. coli* cell lysate.

**Figure S20**. Mass spectra and deconvolution results of the large proteoforms. These results correspond to the proteoforms reported in Figure 4d.

**Figure S21**. (a-f) Deconvolution results of the proteins presented in Figures 4e to 4j.

**Figure S22**. A histogram describing the distribution of the deconvoluted masses (D-score > 20) identified in the analysis of the *E. coli* cell lysate using the double-barrel setup.

# S3. Supplemental tables

**Table S1**. Summary of protein information

| **Proteins** | **Source** | **Abbreviation** | **pI** | **MW (kDa)** | **UniProt ID** |
| --- | --- | --- | --- | --- | --- |
| Carbonic anhydrase | Bovine erythrocytes | CA | 6.6 | 29 | P00921 |
| Amyloglucosidase | Aspergillus niger | Amy | 3.6 | 60-65 | P69328 |
| Trypsin inhibitor | Soybean | TI | 4.5 | 20 | P01070 |
| Bovine serum albumin | Bovine | BSA | 4.7-5.6 | 66 | P02769 |
| Ovalbumin | Chicken egg white | Ova | 4.7 | 44.5 | P01012 |
| Myoglobin | Equine heart | Myo | 7 | 17 | P68082 |
| Ribonuclease A | Bovine pancreas | RNase-A | 9.6 | 13 | P61823 |
| Pembrolizumab | / | Pem | 7.6 | 146 | / |
| Cetuximab | / | Cet | 8.8 | 152 | / |
| Trastuzumab | / | Tra | 9.1 | 148 | / |

**Table S2**. Summary of additional methods used in SCX-UV/FLD

| **MP** | **Mobile phase A** | **Mobile phase B** |
| --- | --- | --- |
| 12 | 40mM AmBc + 20 mM AmAc + 40 mM DFEA + 20 mM MA (pH=5) | 40mM AmBc + 20 mM AmAc + 40 mM DFEA + 20 mM MA (pH=10) |
| 13 | 20mM AmFo + 40mM AmBc + 20 mM AmAc + 40 mM DFEA + 20 mM MA (pH=5) | 20mM AmFo + 40mM AmBc + 20 mM AmAc + 40 mM DFEA + 20 mM MA (pH=10) |
| 14 | 50mM AmFo + 40mM AmBc + 20 mM AmAc + 40 mM DFEA + 20 mM MA (pH=5) | 50mM AmFo + 40mM AmBc + 20 mM AmAc + 40 mM DFEA + 20 mM MA (pH=10) |
| 15 | 50mM AmFo + 20mM AmCa + 20 mM AmAc + 40 mM DFEA + 20 mM MA (pH=5) | 50mM AmFo + 20mM AmCa + 20 mM AmAc + 40 mM DFEA + 20 mM MA (pH=10) |
| 16 | 50mM AmFo + 50mM AmCa + 20 mM AmAc + 40 mM DFEA + 20 mM MA (pH=5) | 50mM AmFo + 50mM AmCa + 20 mM AmAc + 40 mM DFEA + 20 mM MA (pH=10) |
| 17 | 50mM AmFo + 50mM AmCa + 20 mM AmAc + 40 mM DFEA + 20 mM MA (pH=5) | 50mM AmFo + 50mM AmCa + 20 mM AmAc + 40 mM DFEA + 20 mM MA (pH=10) |
| 18 | 20 mM AmAc + 40 mM DFEA + 80 mM MFEA + 20 mM MA (pH=5) | 20 mM AmAc + 40 mM DFEA + 80 mM MFEA + 20 mM MA (pH=10) |

Note: AmAc: Ammonium acetate; AmBc: Ammonium bicarbonate; AmCa: Ammonium carbonate; AmFo: Ammonium formate; DFEA: 2,2-difluoroethylamine; MFEA: 2-fluoroethylamine; MA: Methylamine. The pH of mobile phase A is adjusted by acetic acid and the pH of mobile phase B is adjusted by MA.

**Table S3**. Reported mobile phases used in nanoflow SCX-MS

| **MP** | **Mobile phase A** | **Mobile phase B** |
| --- | --- | --- |
| 19 | 20 mM AmAc (pH=5.6) | 140 mM AmAc + 10 mM AmBc (pH=7.4) |
|  |  |  |

Note: AmAc: Ammonium acetate; AmBc: Ammonium bicarbonate. The pH of mobile phase A is adjusted by acetic acid and the pH of mobile phase B is adjusted by ammonia hydroxide.

**Table S4**. Comparison of three mobile phases used in nanoflow SCX-nMS

| **MP** | **Proteins** | **Intensity** | **Average intensity** |
| --- | --- | --- | --- |
| 11 | CA-Var | 2.64E+05 | 2.18E+07 |
|  | BSA | 2.81E+05 |  |
|  | CA | 5.36E+07 |  |
|  | RNase-A | 3.30E+07 |  |
| 01 | CA-Var | 4.37E+05 | 4.84E+07 |
|  | BSA | 2.17E+05 |  |
|  | CA | 6.08E+07 |  |
|  | RNase-A | 1.32E+08 |  |
| 19 | CA-Var | 1.60E+05 | 8.65E+06 |
|  | BSA | 5.09E+05 |  |
|  | CA | 2.83E+07 |  |
|  | RNase-A | 5.61E+06 |  |

**Table S5**. The m/z values used to extract EICs for protein mixtures

| **Figures** | **Proteins** | **m/z values** |
| --- | --- | --- |
| 3a | CA_Var | 2684.97, 2929.10, 3221.90 |
|  | BSA | 3908.54, 4152.74, 4429.54, 4777.33 |
|  | CA | 2909.69, 3232.89 |
|  | RNase-A | 1955.46, 2281.42, 2737.38, 3041.40, 3421.53 |
| S12a | CA_Var | 2684.94, 2932.45, 3221.83 |
|  | BSA | 3910.63, 4157.25, 4436.79 |
|  | CA | 2909.73, 3232.90 |
|  | RNase-A | 1955.43, 2281.59 |
| S12c | CA_Var | 2684.77, 2929.17, 3222.02 |
|  | BSA | 3908.50, 4154.74, 4431.68 |
|  | CA | 2909.76, 3232.98 |
|  | RNase-A | 1955.73, 2281.19, 2737.35, 3041.41 |

**Table S6**. The m/z values used to extract EICs for Figures 4b and 4c.

| Figures | IEC Mode | m/z values |
| --- | --- | --- |
| 4b | SAX | 2270.31, 2497.26, 2598.49, 2940.69 (1); 2111.93-2168.46, 2792.11, 2944.11, 3141.19 (2); 2792.11, 2944.11, 3141.19, 3402.75 (3); 1782.76, 1894.22, 2020.46, 2164.62 (4); 2237.27, 2523.23, 2754.71, 3030.13, 3237.91 (5); 3790.70, 4001.28, 4236.58, 4501.36 (6); 3174.59, 3439.03 (7); 2928.63, 3153.80 (8); 2661.45, 2809.52, 3043.60, 3242.71 (9); 2023.05, 2275.81, 2600.83 (10); 3896.56, 4156.18, 4449.29 (11); 3013.02, 3213.79, 3433.25, 3708.10 (12); 1215.47, 1244.42, 1272.38 (13); 2000.51, 2199.94 (14); 3346.75 (15); 1624.61, 1948.75 (16); 4777.95, 5096.28, 5460.44 (17); 4885.78, 5107.75, 5350.99 (18); 1300.15, 2460.71 (19); 1516.57 (20); 5085.89, 5289.37, 5509.73 (21). |
| 4c | SCX | 3260.65, 3532.26 (1); 3710.54 (2); 2940.65, 3234.61 (3); 2579.18, 3829.18, 4104.21 (4); 2834.60, 3121.76 (5); 2898.89, 3220.86 (6); 4934.24, 5178.44, 5450.31 (7); 2204.93, 2359.46, 2519.44 (8); 3842.18, 4086.03 (9); 3153.76, 3416.52, 3708.03 (10); 5034.18-5068.97 (11); 3323.40, 3600.30 (12). |

**Table S7**. Collections of the average time range used for Figures 4e to 4l.

| Figures | IEC Mode | Average time range (min) |
| --- | --- | --- |
| 4e | SAX | 14.02-14.36 |
| 4f | SAX | 8.73-9.07 |
| 4g | SAX | 9.70-10.09 |
| 4h | SAX | 13.16-13.56 |
| 4i | SAX | 23.16-24.01 |
| 4j | SAX | 33.22-34.47 |

# S4. CRediT authorship contribution statement

**Ziran Zhai**: Conceptualization, Methodology, Investigation, Writing - Original Draft, Writing - Review & Editing. **Hafsa Zakri**: Methodology, Investigation. **Matteo Damian**: Methodology, Investigation, Resources. **Francesco G. Mutti**: Methodology, Resources. **Garry Corthals**: Writing - Review & Editing. **Andrea F.G. Gargano**: Conceptualization, Methodology, Investigation, Project administration, Supervision, Resources, Writing - Original Draft, Writing - Review & Editing.

# S5. References

(1) Zhai, Z.; Mavridou, D.; Damian, M.; Mutti, F. G.; Schoenmakers, P. J.; Gargano, A. F. G. Characterization of Complex Proteoform Mixtures by Online Nanoflow Ion-Exchange Chromatography-Native Mass Spectrometry. *Anal Chem* **2024**, *96* (22), 8880-8885.

(2) Yan, Y.; Liu, A. P.; Wang, S.; Daly, T. J.; Li, N. Ultrasensitive Characterization of Charge Heterogeneity of Therapeutic Monoclonal Antibodies Using Strong Cation Exchange Chromatography Coupled to Native Mass Spectrometry. *Anal Chem* **2018**, *90* (21), 13013-13020.
